# Digitalizing informed consent in healthcare: a scoping review

**DOI:** 10.1186/s12913-025-12964-7

**Published:** 2025-07-02

**Authors:** Mascha Goldschmitt, Patricia Gleim, Sekina Mandelartz, Philipp Kellmeyer, Thomas Rigotti

**Affiliations:** 1 Department of Work, Organizational, and Business Psychology, Johannes Gutenberg University Mainz, Wallstr. 3, Mainz, 55122 Germany; 2 Department of Neurosurgery, University of Freiburg– Medical Center, Breisacher Str. 64, Freiburg I.B, 79106 Germany; 3 School of Business Informatics and Mathematics, University of Mannheim, B6, 26, Mannheim, 68159 Germany; 4 Leibniz Institute for Resilience Research (LIR) gGmbH, Wallstr. 7, Mainz, 55122 Germany

**Keywords:** Digital consent, Informed consent, Patient information, Digitalization, Telemedicine, Medical research

## Abstract

**Background:**

Traditional paper-based informed consent for medical procedures poses a number of challenges, such as low comprehensibility, lack of customization, and limited time for discussion with medical staff. Digitalization, especially in light of the rapid development of AI-based technologies, could provide a solution.

**Methods:**

This scoping review explores the digitalization of the consent process, focusing on the types of technologies used, their role in the consent process, evaluation results, and success factors for implementation. Following the guidance of the Joanna Briggs Institute (JBI) Manual for Evidence Synthesis for scoping reviews, we searched various databases and platforms (Web of Science, EBSCOHost, PubMed and PubPsych) for eligible articles published between January 2012 and June 2024.

**Results:**

Title and abstract screening of 4287 records resulted in the inclusion of 27 studies for analysis. The findings suggest that digitalizing the consent process can enhance recipients' understanding of clinical procedures, potential risks and benefits, and alternative treatments. Mixed evidence exists on patient satisfaction, convenience, and perceived stress. The limited research on healthcare professionals indicates that time savings are the major benefit. AI-based technologies seem to be not yet suitable for use without medical oversight.

**Conclusions:**

Overall, few interactive technologies have been evaluated in the patient consent process, and only recently have studies started to examine the use of AI technologies. This indicates an early stage of the digitalization of patient consent for medical diagnosis and treatment. However, there is great potential to optimize the consent process for both patients and healthcare professionals. Methodologically sound studies are needed to validate these findings.

**Trial registration:**

The scoping review was initially preregistered with PROSPERO (CRD42023397681) as a systematic review. The reasons for the change to a scoping review are outlined in the registration, while the systematic approach to data extraction and analysis was maintained.

**Supplementary Information:**

The online version contains supplementary material available at 10.1186/s12913-025-12964-7.

## Background

Informed patient consent is the act of a patient willingly agreeing to a medical procedure and has traditionally relied on paper-based methods. In contrast to other areas of health care, such as disease diagnosis [1–3], the digitalization of this process has only recently received increased attention. However, digitalizing the informed consent process might offer patients and healthcare professionals remarkable opportunities, but legal regulations, data protection as well as ethical aspects need to be considered. Especially in light of the rapid advances in artificial intelligence (AI), it is essential to critically assess the potential of the different technologies to enhance the consent process for the benefit of those involved.

Even before their upcoming treatment, patients seek information about their disease, treatment, and possible risks [4, 5], often turning to the Internet for health information [6]. Proactively seeking answers on the Internet may be risky, however, as numerous studies have shown the inadequacy of publicly available information on various diseases or treatments in terms of quality as well as the required reading level or health literacy [7, 8]. In light of the advancements in publicly available generative AI, it was found that compared to Google, ChatGPT (OpenAI; San Francisco, USA) has the potential to provide more valuable answers to patients' questions, but it is not yet a reliable alternative, as there is still a risk of incomplete or misleading information being provided [9]. There is a need for reliable, comprehensible, and easily accessible health information [10], which could improve patients’ preoperative satisfaction [11]. For these and legal reasons, it seems evident that healthcare providers themselves should refer or provide adequate information to their patients [12]. However, delivering high-quality information alone does not ensure patient understanding.

Research indicates that traditional consent forms are often too complex [13], leading to uninformed consent as patients may not read them thoroughly [14–16]. Patients and their families prefer oral preoperative information [16], which leads to better understanding than written informed consent forms [17]. Yet, physicians frequently underestimate patients’ need for detailed preoperative information [18] and struggle to provide standardized yet individualized information, considering factors such as age, education [19], individual preferences for information [20, 21], or language barriers [22]. It is questionable whether physicians’ communication skills and the limited time available for educational interviews can meet these demands. During the COVID-19 pandemic, physicians have often been overwhelmed with clinical duties, pushing communication to a lower priority [23]. Overcrowding reduces the amount of time physicians can spend per patient, and pre-existing time constraints – due to factors like staffing shortages or administrative burdens – can also contribute to inefficient patient throughput, longer wait times, and repeated visits, thereby exacerbating overcrowding [24]. The shortage of physicians’ time with patients might lead to time pressure [25], a job stressor that has been identified as a significant predictor of emotional exhaustion [26], oncologist burnout, and compassion fatigue [27] and is associated with a lack of control, stress, and the intent to leave [25]. The use of AI in diagnostics has been shown to alleviate time pressure on physicians, suggesting potential benefits for the consent process [28]. There is growing interest in developing technology-enabled tools, such as chatbots, to assist patients in understanding medical procedures and consenting to them [29]. Studies have shown positive findings on the acceptability of chatbots in conveying information to patients [30], a preference of chatbot responses to patient questions in a social media forum over physician responses [31], and time savings for clinicians [32]. However, AI-generated patient information often still lacks consistent reliability and requires professional oversight [33, 34]. To ensure that the needs of patients continue to be considered with the implementation of AI, high-quality patient involvement is needed [35]. The acceptability of AI-based consent forms remains to be investigated.

Digitalizing the informed consent process, particularly through artificial intelligence, offers significant potential for improving doctor-patient communication. However, its application in the consent process requires careful consideration of a wide range of skills and competencies [36] due to potential risks, such as measurement error, selection bias, and feedback loop bias [37]. To our knowledge, this scoping review is the first to consolidate the growing body of research on digital and AI-based tools in the consent process for medical examinations and treatments, with a focus on their impact on both patients and practitioners. We aim to address three key research questions (RQs):

1. Which technologies are used to digitalize the informed consent process for medical examinations and treatments, and what role do they play in this process?
2. What are the evaluation results regarding domains, user groups, and differential effects?
3. What are the success factors for implementing such systems?

Domain is used here to refer to a specific outcome dimension assessed by the primary studies, e.g., patient comprehension, patient satisfaction, affective responses (stress/anxiety), knowledge retention, convenience/usability, or clinician-centered outcomes such as time savings. Differential effects refer to variations in outcomes between different subgroups, such as younger vs. older patients, individuals with low vs. high health literacy, or caregivers vs. patients. This review will aid researchers in exploring the interaction between technology and healthcare and guide the development of suitable technologies. Practitioners can learn about the advantages of digital consent processes for patient care and workflow efficiency. Ultimately, this review contributes to digital health research and aims to spur thoughtful discussions on the use of technology to improve informed consent and enhance healthcare workflows.

## Methods

### Search strategy and selection criteria

This review was prospectively registered as a systematic review with PROSPERO (CRD42023397681) [38]. As the small number of suitable studies limited the ability to provide quantitative answers to our research questions, which are essential for a systematic review and meta-analysis, we adopted a scoping review approach, as recommended for areas where the evidence is emerging and not yet amenable to systematic review [39]. Scoping reviews are intended to map the breadth and depth of evidence on a particular topic without necessarily focusing on the critical appraisal of the included studies [39, 40]. To ensure rigor and transparency in our review process, we maintained a systematic approach following the guidance of the Joanna Briggs Institute (JBI) Manual for Evidence Synthesis for scoping reviews [41]. Furthermore, this review was reported following the Preferred Reporting Items for Systematic Reviews and Meta-Analyses extension for Scoping Reviews (PRISMA-ScR) guidelines [42].

Based on Methley et al.’ s [43] comparison of PICO(S) and SPIDER, we chose the PICO framework to define the search string and structure the eligibility criteria (Table 1). While other frameworks, such as PCC, may also be appropriate for scoping reviews depending on their scope, we found PICO particularly helpful for maintaining conceptual clarity in our approach. The search incorporated general health-related keywords, keywords related to the outcome, and digital interventions in the patient education process. We did not include search terms for the comparison category to avoid unnecessarily narrowing the search results. Owing to the recent development of AI, the expected small number of empirical studies on its impact to date, and the assumed transferability of findings from non-AI-based to AI-based technologies, the study was not limited to the use of AI systems but considered web-based or app-based technologies in general. Radiology was included in the search string as it is a medical specialty where digitalization and AI-supported tools are particularly advanced, making it a relevant field for identifying developments in the digitalization of patient consent. Innovations in radiology may serve as early indicators or models for broader applications of digital consent processes. Preliminary searches revealed many studies on the readability of patient information and general patient education. To ensure alignment with our specific focus on the digital enrichment of informed consent for medical examinations and treatments, related keywords were explicitly excluded.

**Table 1:** Search string used in scientific databases following PICO search tool

	Search string
Population	(health OR “public health” OR “health care” OR healthcare OR “health service” OR “health sector” OR radiology OR “clinical radiology” OR “interventional radiology”)
	AND
Intervention	(chatbot OR “dialogue system” OR “dialog system” OR “digital communication system” OR “digital communication assistant” OR “voice assistant” OR “conversational assistant” OR SIRI OR Alexa OR “Google Assistant” OR “IBM Watson” OR “artificial intelligence” OR AI OR web-based OR online OR NLG OR “natural language generation” OR NLP OR “Natural language processing” OR NLU OR “Natural language understanding”)
	AND
Comparison	[not applicable]
Outcome	(„patient information “ OR (consent AND patient)) NOT (education AND readability)

Using the described search string with Boolean operators, the following databases or platforms were searched: Web of Science, EBSCOHost (including APA PsycArticles, APA PsycInfo, CINAHL, Communication Abstracts, eBook Collection, eBook Open Access Collection, MEDLINE, OpenDissertations, PSYNDEX Literature with PSYNDEX Tests), PubMed, and PubPsych. A detailed description of the search methods and information sources following PRISMA-S [44] can be found in the additional information to this article (see Additional file 1).

### Inclusion and exclusion criteria

We included empirical studies (quantitative, qualitative, and mixed-methods designs), reviews, and meta-analyses published in peer-reviewed journals. Grey literature (e.g., unpublished reports, dissertations, government documents) was not included to focus on sources with transparent and peer-reviewed methods. Due to the rapid development of technologies and preliminary test searches indicating an increasing number of relevant articles in recent years, we limited the publication date to the period between 1 January 2012 and 18 June 2024. Studies were eligible if they focused on the implementation or evaluation of web-based, app-based, or AI-supported interventions in the context of informed consent processes in routine clinical care. We excluded publications that did not report outcomes (e.g., protocols) or focused solely on the technical implementation of systems. We also excluded literature on consent for clinical trial participation, as we assume that the emotional and administrative context differs from routine clinical care. Patients undergoing medical procedures often face immediate health-related risks and time-sensitive decisions, which may involve different psychological dynamics than consent to participate in research, where personal risk is typically minimal. Moreover, obtaining informed consent in routine care must be integrated into everyday clinical workflows, whereas studies are time-limited and may rely on dedicated administrative resources. We excluded publications in languages other than English or German, as we could not ensure accurate interpretation of their content. This was primarily done during title and abstract screening; only one article was excluded at the full-text screening stage due to the full text being available in Spanish only.

### Selection process

After duplicate removal, two reviewers independently screened titles and abstracts using Rayyan [45] for blind screening. Disagreements were discussed among all five reviewers, leading to a joint decision on inclusion or exclusion. Articles deemed potentially relevant based on title and abstract were retrieved in full text and assessed for eligibility by one reviewer. Uncertainties were discussed within the review team to reach a consensus. Only those studies that fulfilled the inclusion criteria after full-text screening were included in the final analysis.

### Data extraction and synthesis

A structured extraction spreadsheet was drafted in January 2023 by two reviewers (MG and PG) in consultation with the full author team and was piloted on five randomly selected full-text articles to ensure clarity and consistency, leading to minor wording adjustments. The final version, shown verbatim in Additional file 2, includes columns for study characteristics, technology type, setting, sample, outcome domains, user group, reported effects, and implementation facilitators or barriers.

The extraction strategy was aligned with the three research questions guiding this review. To address RQ1, we collected general study information, a detailed description of the digital technology used in the consent process, and, where applicable, specifics of the intervention. For RQ2, we recorded which outcome domains each study evaluated, the user group concerned (patients, caregivers, or clinicians), and whether the reported effects in each domain were positive, neutral, or negative. Accordingly, 'success' was captured exactly as articulated in each primary study – whether patient-centered (e.g., improved comprehension or satisfaction), clinician-centered (e.g., reduced consultation time), or organizational/system-centered (e.g., fewer procedure cancellations). RQ3 was addressed by extracting all statements referring to implementation facilitators or barriers, which were then inductively categorized into overarching themes such as user-centered design, workflow integration, or legal compliance.

Additionally, relevant findings that did not fall within predefined categories but were considered informative for answering the research questions were documented. The synthesis of results followed the structure of the three research questions, which also guided the organization of the findings section.

## Results

**Fig. 1:**
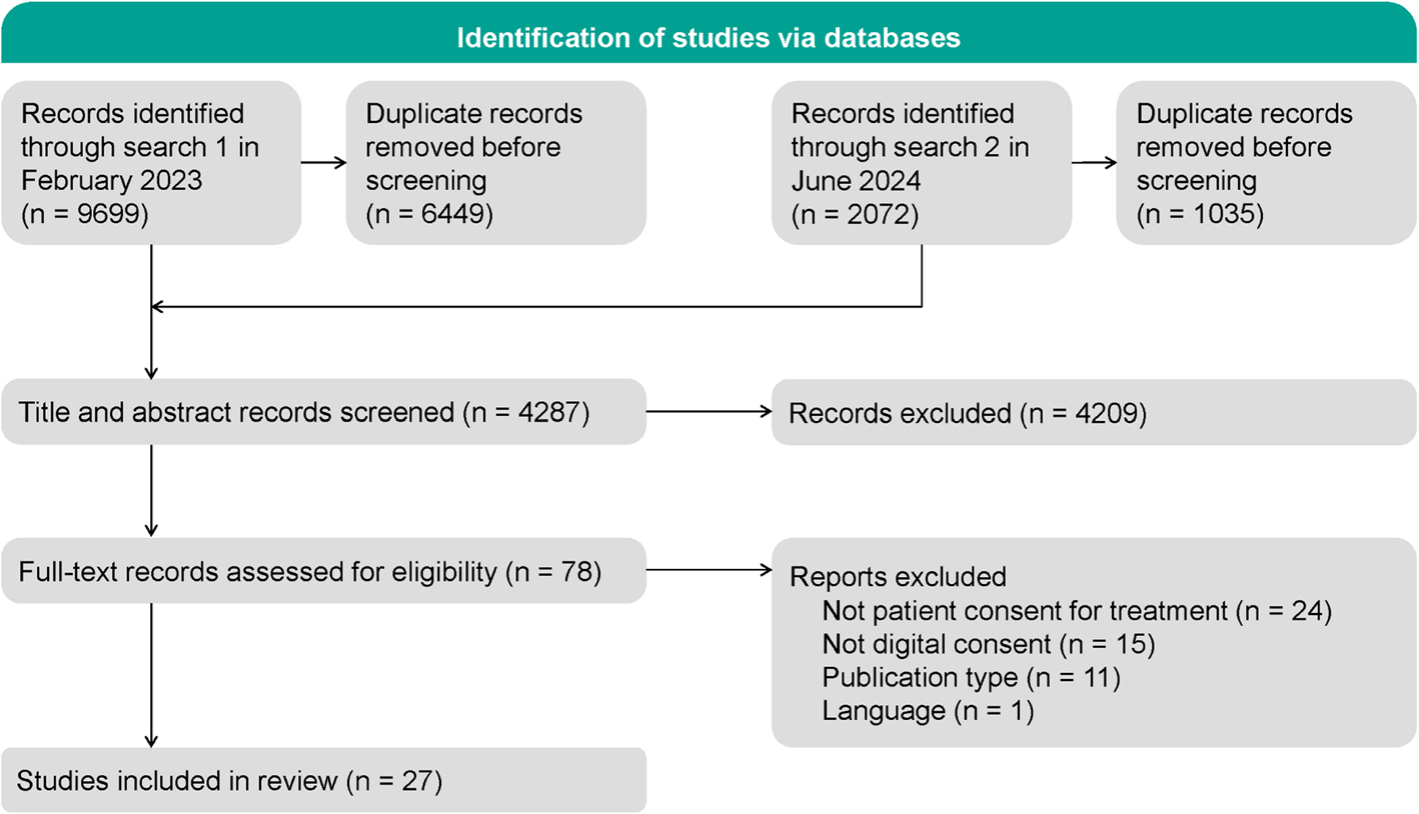
PRISMA-ScR flow diagram of study selection

The initial literature search was conducted in February 2023 and updated in June 2024. The screening process, including exclusions, is outlined in the PRISMA-ScR diagram (see Fig. 1). From the electronic database search, a total of 11771 studies were retrieved, with 7484 duplicates removed. After title and abstract screening of 4287 records, 78 full-text articles were screened for eligibility, yielding 27 studies for data extraction and qualitative synthesis.

The characteristics of the 27 included studies are summarized in Additional file 2. Key insights from the included articles are presented below, organized by the three research questions.

### Research question 1: Which technologies are used to digitalize the informed consent process for medical examinations and treatments, and what role do they play in this process?

In addressing our first research question on technologies for digitalizing the informed consent process, we present our findings in a structured manner. We begin with studies where the applied technology was not clearly specified or referred broadly to telemedicine. Thereafter, we examine distinct technological approaches, arranged in ascending order based on their interactivity and adaptability within the consent process. Table 2 provides a concise overview of the types of technologies used across the included studies, serving as an entry point for the more detailed discussion that follows.

**Table 2:** Frequency of technology types used in the 27 included studies

Technology type	No. of studies	% of total	References	Typical role in consent process
Technology not specified or telemedicine in general^*^	7	26%	[46–52]	End-to-end remote or hybrid consent
Video	6	22%	[53–58]	Supplement or partially replace verbal explanation
Website	2	7%	[59, 60]	Self-paced content before face-to-face visit
Digital questionnaire	1	4%	[61]	Pre-operative data capture feeding into consent
Interactive web application	3	11%	[62–64]	Personalized walkthrough plus clinician alerts
AI	8	30%	[65–72]	Interactive Q&A or automated document generation
Total	27	100%	—	—

^*^Includes studies that discussed telemedicine consent broadly or evaluated digital consent workflows without specifying a single tool

#### Technology not specified or telemedicine in general

Beyond specific technologies, several studies addressed the general digitalization of the informed consent process or evaluated telemedicine-based approaches. Digital consent solutions have been explored as a means to facilitate and streamline the informed consent process by enhancing communication, improving documentation practices, and optimizing the structure and efficiency of consent discussions [46–48]. Nevertheless, implementation remains limited, possibly due to technical infrastructure limitations or compatibility issues with existing systems [46]. Legal acceptance of online consent remains limited in many European countries, due to concerns about the absence of direct patient observation and unclear regulatory frameworks governing digital consent procedures. To address these issues, the implementation of secure, data-protected services, appointment scheduling, QR code-based identity verification, explicit parental consent in pediatric cases, and the option to revoke consent before procedures has been recommended [46, 50]. Patient perspectives across various studies indicated a general willingness to provide digital consent, although preferences were context specific [47]. Essential patient requirements included clear, understandable information about data usage and protection [47]. The appropriateness of digital consent technologies appears to vary depending on the medical procedure and patient characteristics, underscoring the need for transparent and individualized consent processes [47, 48]. Finally, telemedicine can be especially useful for high-risk patients and offers potential to mitigate barriers related to travel, childcare, and time constraints [50, 52]. Providing remote consent options as an alternative to in-person processes could better accommodate patient preferences and improve access to care [52].

#### Video

Six studies investigated the use of standardized videos to support the informed consent process. These videos were typically designed to illustrate specific procedures, such as blood transfusion [55], lumbar puncture [53], radiotherapy [56], or hernia surgery in children [57]. Their main purpose was to complement or partially replace the verbal explanation provided during the consent conversation. From a clinical perspective, videos served as a preparatory resource to support interactive discussions between patients and healthcare professionals [58]. Compared to traditional written materials, they offered a more accessible format, allowing patients to engage with the content at their own pace [58]. Videos were particularly designed to support patient groups with cognitive or sensory impairments [53], or legal guardians responsible for providing consent on behalf of children [57]. In terms of design, studies emphasized the importance of clarity, simplicity, and visual illustration [56, 58]. While some patients appreciated the detailed information provided [56], others suggested reducing complexity and including more visual elements or actors [55]. Additional design recommendations included adding subtitles to improve accessibility and enabling external access to videos for sharing with family members [56].

#### Website

Expanding on the use of videos, two studies examined websites that incorporate similar audiovisual elements, such as animations, while offering more interactivity and flexibility in how patients access the information. In both randomized controlled trials, the digital content served to provide patients with more information than is available through traditional face-to-face discussions with doctors. The platforms provided structured, self-paced modules, either as text-based webpages [59] or as video-based slide presentations [60], covering key information such as risks, benefits, and procedural expectations. The content was designed to be comprehensible and user-friendly, with one study recommending a reading level equivalent to the fifth grade to ensure accessibility [59]. Web-based applications were not intended to replace personal physician–patient interaction, but rather to standardize and streamline the delivery of core information. Additionally, they allowed flexible timing, giving patients the opportunity to engage with the material in advance of the consent conversation.

#### Digital questionnaire

In addition to interactive technologies, other digital tools can assist in the informed consent process by enhancing data collection and improving workflow efficiency. One such tool is the ePAQ-PO (electronic Preoperative Anesthetic Questionnaire), a web-based assessment developed by Goodhart et al. [61]. This tool is completed by patients prior to surgery and gathers relevant medical data, such as body mass index (BMI) and physical status. The initial validation with 300 patients showed that the data collected through the ePAQ-PO was consistent with clinician-assigned values and was well-received by patients. While the ePAQ-PO does not directly capture the consent process, it collects relevant patient data before medical treatment, suggesting potential benefits for the digital consent process in terms of efficiency and accuracy.

#### Interactive web application

Interactive web applications represent a more advanced and personalized approach to digital informed consent by combining multimedia content with interactive features. These tools typically allow patients to explore consent information at their own pace and can incorporate quizzes, glossaries, or communication functions to enhance comprehension and engagement [62–64]. The design of these applications emphasizes clarity, completeness, and user-centered functionality. Key recommendations across the studies include using scripted, comprehensive content that can be tailored to individual risk profiles [62], offering interactive elements such as quizzes to reinforce understanding [62], and enabling patients to flag confusion or concerns directly within the platform [63, 64]. For example, features like emoticon-based feedback buttons can trigger follow-up by doctors (if *I don’t understand this*) or nurses (if *I feel worried*), facilitating communication [63]. Usability testing also highlights the importance of intuitive design, role-specific interfaces, and adjustable language levels to match the needs of both patients and healthcare professionals [64].

#### AI

A recent technological development in the field of digital informed consent is the use of AI-driven chatbots and applications powered by large language models (LLMs) such as ChatGPT. These tools can either guide patients interactively through the consent process or support the generation and simplification of educational materials and consent documents. As the only study investigating an AI-based solution before the publication of ChatGPT, Schmidlen et al. [65] evaluated a rule-based chatbot designed to guide patients through the consent process, offering additional information upon request. Users appreciated the ease of use and the ability to control the pace and depth of the interaction, favoring the system’s structured, dialogue-based format. More recent studies have explored the use of ChatGPT for both the automated simplification of existing consent forms [66, 67] and direct interaction with patients by answering their individual questions [69–71]. These applications utilized different versions of the model (GPT-3.5 and GPT-4) and often compared AI-generated content with traditional consent materials or verbal explanations by physicians.

In the context of document simplification, ChatGPT-4 consistently produced medically and legally sufficient content, while improving readability, reducing technical complexity, and lowering passive voice frequency [66]. However, board-certified professionals were generally more critical than nonmedical users regarding the accuracy and informativeness of AI-generated content [67].

When used in interactive scenarios, ChatGPT’s outputs were typically rated as understandable and medically accurate, although important limitations emerged. GPT-3.5 was considered plausible but occasionally incomplete or factually incorrect – limitations that could pose a risk to patients due to misinformation [68, 71]. While GPT-4 demonstrated higher precision and greater content depth, it still exhibited some gaps, highlighting the need for professional oversight [68]. A common criticism was the lack of visual aids and the occurrence of fabricated references produced by AI systems. Kienzle et al. [69], for instance, found that only a minority of the citations generated by the evaluated chatbot were accurate and relevant.

In a randomized controlled trial by Aydin et al. [72], providing informed consent by asking GPT-3 questions was directly compared to a conventional physician-led consent process for coronary angiography. ChatGPT-based consent was found to be equally accurate and comprehensive, with participants in the chatbot group showing a better understanding of treatment risks. The ability to ask personalized questions and process information at their own pace may have contributed to this improvement.

Despite these promising results, important limitations persist. Although lack of familiarity with such technologies was not a barrier to adoption [65], the information provided by ChatGPT was sometimes overly general and assumed prior medical knowledge, potentially limiting accessibility for patients with lower health literacy [70]. While the personification of the chatbot was perceived as positive [65], the lack of graphical presentation was also seen as a disadvantage, particularly for conveying complex medical information in an accessible way [70]. In addition, patients expressed a clear preference for human interaction in critical decision-making contexts, citing concerns about source reliability and trust [71]. Echoing this, two studies emphasized that AI-based systems should complement, rather than replace, face-to-face consultations [65, 67]. In conclusion, while AI-driven tools – especially advanced models like GPT-4 – show promise as preliminary resources or support systems in the consent process, they are not yet reliable enough to fully replace human interaction or expert review.

### Research question 2: What are the evaluation results regarding domains, user groups, and differential effects?

For our second question on the evaluation results regarding domains, user groups, and differential effects, the main domains include patient understanding, patient satisfaction (including perceived stress), knowledge retention, convenience of use, and time savings for healthcare professionals.

Traditional consent methods have been found to negatively impact patient engagement and understanding. For instance, Cheung et al. [55] found that traditional consent discussions without video led to inconsistent patient recall, with many seeking additional information online, while Robertson et al. [58] noted that most patients and caregivers felt overwhelmed, hindering engagement with written materials. In contrast, most studies assessing digitalized consent processes highlight positive impacts on patients’ or proxy decision-makers’ (such as parents) understanding of the planned clinical procedures [48, 53, 54, 56, 57, 59, 60, 65], potential risks and benefits [54, 55, 72], and alternative treatments [55, 62]. There is mixed evidence on whether digital training can improve knowledge retention over time [49, 60]. Of the studies that assessed the impact of digitizing informed consent on patient satisfaction, all but two reported a significant increase in satisfaction [48, 52, 53, 59]. The remaining two studies found no change in satisfaction [60, 72]. Some evidence also suggests that digital consent tools may help reduce patient stress levels [50, 56, 57]. However, disclosing uncertainties through digital formats can increase anxiety for some patients [63]. This effect may be mitigated by well-designed systems that combine clear explanations, emotional reassurance, and human support, thereby promoting meaningful and informed decision-making. The evidence on the convenience of different technologies for digitalizing informed consent is mixed. On the one hand, asking questions via a digital channel can facilitate the information and consent process [52, 63], with AI-based technologies in particular enabling a more individualized and therefore possibly more appropriate response to patient questions [72]. It also seems to be perceived as comfortable to be able to control the time and speed of the consent process [65]. On the other hand, some technologies did not affect patients’ comfort during the consent process [55]. The heterogeneity of technologies and applications makes it challenging to gauge the overall impact of digitalized consent on patients. With respect to different user groups, patient opportunities for digital consent seem to lie mainly in improving the understanding of diagnostic and therapeutic procedures, providing more individualized information, and reducing patient barriers such as travel, childcare, sensitivity of medically high-risk patients, or language barriers.

The impact of digital consent on healthcare professionals was less frequently mentioned, but time savings, partly attributed to reduced case cancellations [49], emerged as the most commonly reported benefit [46, 60]. Digital consent also has the potential to increase quality and reduce costs related to consent claims [54]. For example, using a mobile app for preoperative instructions and reminders has been shown to improve patient compliance and reduce cancellations [49]. However, implementing new technologies into existing workflows was deemed challenging by practitioners [63] and could lead to additional workload [64]. Not only the initial integration of new technologies, but also the digitalized and thus more individualized consent could, in addition to the advantages for patients described above, also lead to additional workload. The possibility of asking questions or uncertainties to healthcare practitioners in the digitalized application could lead to a higher burden on them [63]. However, this additional time might be offset by future process simplification [46, 64]. While initial expenses are needed, digital consent may reduce economic costs [46], for example through improved efficiency and accuracy [50, 61], fewer lost consent forms [64], and safer storage and preservation of consent forms [46].

While digital consent technologies offer numerous advantages and address several challenges of traditional consent processes, a fully digitalized approach does not appear to be universally desired or appropriate. Some patients continue to express a preference for face-to-face interactions, and healthcare professionals likewise emphasize the value of personal communication during the consent process [51, 71]. Therefore, digital solutions should be implemented to complement and enrich, rather than replace, direct patient-physician interactions. By enhancing personal communication with digital tools, the consent process can be optimized to support better understanding, trust, and patient engagement without compromising the essential human connection.

### Research question 3: What are the success factors for implementing such systems?

Our third research question concerns factors for successfully implementing digital consent technologies. While the current body of literature is methodologically and qualitatively limited, several critical success factors can be identified.

#### Patient-centric approach and usability

One of the core elements of successful implementation is ensuring that technology positively impacts the patient experience. This requires designing systems that improve comprehension of procedural details, potential risks and benefits, and alternative treatments. Reducing barriers to accessing and understanding information, for example with regard to different technological literacy, is essential to accommodate diverse user needs [63–65]. Systems should be intuitive, user-friendly, and allow patients to control the timing and pace of the consent process [65, 72]. Additionally, transparency regarding data privacy and use, as well as the ability to customize the consent process, are important to foster trust and ensure patients feel confident in the system [50, 64, 65]. End-user involvement during the development phase is considered essential for acceptance and successful implementation [64]. Moreover, it is essential to ensure that patients are fully aware of the options available to them during the consent process to ensure informed decision-making [52].

#### Consistency of information

Ensuring alignment between digital content and face-to-face discussions is paramount to building trust in the information provided. Any discrepancies between the two mediums could undermine patient confidence in the consent process and the information being presented. AI tools, such as ChatGPT, show potential for simplifying language and improving accessibility, but they must be integrated with care to avoid issues with the accuracy and consistency of information [63, 65, 67–71]. Especially when working with AI, the accuracy of the information often cannot be guaranteed [67–71]. Therefore, care should be taken to ensure that the stored database that the AI accesses is reviewed and, where possible, limited. The use of AI in patient education requires medical oversight to ensure the accuracy of the information provided to patients [68, 71].

#### Integration with existing clinical workflows

Seamless integration of digital consent systems into current clinical workflows is critical. However, this requires careful planning to minimize disruptions. Challenges include ensuring legal validity and maintaining consistent information across formats. Several studies emphasized that AI tools should complement rather than replace face-to-face consultations [65, 67]. It is also important that healthcare providers and patients are sufficiently trained, with support systems in place to address any concerns or issues that may arise during implementation.

#### Legal compliance

Adherence to existing and forthcoming regulations, particularly those concerning data privacy and security, is crucial for the successful implementation of digital consent technologies. Neumann et al. [51] highlighted that the adoption of remote consent is limited in many European countries due to inconsistent legal and technical structures. This challenge is especially pronounced in regions with lower gross domestic product per capita, where improving technical standards could expand digital consent usage and benefit patients. Additionally, the upcoming EU AI Act introduces new requirements for the integration of AI tools into clinical practice, which must be considered to ensure legal compliance. The lack of clarity regarding legal requirements in certain regions further underscores the need for a unified and clear legal framework to facilitate the widespread adoption of digital consent systems [51].

#### Cost-effectiveness

While the initial investment in digital consent systems may be high, their potential to reduce long-term costs is a compelling argument for their implementation. Potential savings include improved efficiency, fewer lost consent forms, and safer storage of consent-related data. In addition, digital systems may help reduce the costs associated with consent-related claims by improving accuracy and documentation quality [54]. Over time, the initial costs may be offset by simplified workflows and increased operational efficiency [46, 50, 61, 64].

In summary, successful implementation of digital consent technologies hinges on ensuring patient-centric design, ensuring usability, aligning digital content with face-to-face discussions, integrating new technologies with existing workflows, adhering to legal and regulatory standards, and ensuring long-term cost-effectiveness. While challenges remain, especially concerning legal requirements and user diversity, addressing these factors can pave the way for effective and widespread use of digital consent systems in healthcare.

## Discussion

### Summary and discussion of main findings

This scoping review identified a diverse array of digital technologies employed to enhance the informed consent process in healthcare settings. These range from standardized videos and interactive web applications to AI-driven chatbots and LLMs. Our results suggest that the digitalization of informed consent can enhance patient understanding, streamline the consent process, and improve accessibility by providing patients with more comprehensible and individualized information. Here, patients found the control over the speed and timing of the information phase as well as animations and graphics to be particularly helpful. These findings align with broader research showing that digital and AI-based tools can improve the patient journey by supporting personalized care, enhancing diagnostics, and reducing wait times [73, 74]. AI has also been recognized for its potential to improve patient safety and clinical decision-making when effectively implemented [75]. Of the studies we analyzed, only a few investigated the impact of digitized consent on healthcare staff, primarily identifying time savings as benefits. With regard to the different technologies, AI-based tools in particular seem to hold promise in individualizing the information and consent process by communicating directly with the patient or generating consent documents adapted to their language or reading level. However, the use of these technologies still requires human oversight, as even advanced LLMs may produce inappropriate or inaccurate content. In addition to potential inaccuracies in medical information, the integration of AI into the patient journey introduces further risks that warrant critical consideration. One pressing concern is the perpetuation of bias through large language models. Recent studies show that LLMs can perpetuate harmful, ethnicity-based medical misconceptions [76] or reproduce structural healthcare disparities, projecting more favorable outcomes for white patients and recommending unequal treatments [77]. Beyond racial bias, growing evidence highlights additional forms of algorithmic distortion – including gender-related and data-driven biases – that stem from unbalanced training datasets and limited demographic representation [78]. Crucially, these biases are not only embedded in the data itself but also reflect structural inequalities in how clinical AI research is produced. Studies show that datasets and authorship are disproportionately concentrated in high-income countries, particularly the United States and China, with leadership roles often occupied by men from a limited range of specialties [79]. These dynamics risk embedding narrow perspectives in AI systems, compromising diagnostic accuracy and reinforcing inequities in clinical decision-making. Addressing this requires greater diversity not only in data but also in the institutions and voices shaping AI development. This underscores the importance of evaluating AI tools thoroughly before clinical implementation. In response to these disparities, the collection of diverse demographic and clinical data has been proposed to enhance AI performance and ensure broader representation. As Fiske et al. [80] argue, such data can enhance algorithmic accuracy and support more equitable diagnostic outcomes, though it also introduces new ethical challenges, including privacy risks and the potential misuse of sensitive data. Thus, while data diversification may mitigate certain biases, it must be carefully balanced against concerns of trust, transparency, and data governance. As the responsible collection and use of sensitive demographic data become more central to AI development, questions about data control, protection, and patient comfort in sharing it remain crucial. This aligns with empirical findings showing that patients express a broad range of concerns, including worries about safety, loss of personal choice, increased costs, data-source bias, and data security. Importantly, patient support for AI is often conditional on whether these risks are transparently addressed [81]. These concrete concerns are embedded within broader trust dynamics. Recent research shows that individuals' preferences for AI in healthcare are shaped by both cognitive and affective trust mechanisms. While perceived benefits influence openness to AI tools, risk perceptions – especially regarding opaque decision-making and potential harms – carry greater weight in shaping resistance [82]. Notably, implicit trust tends to favor physicians over AI, particularly when potential risks are perceived as high. This corresponds with indications that fully digital approaches may not be equally acceptable to all patient groups. Some studies suggest that certain patients may prefer face-to-face consultations [51, 71], and physicians likewise voiced concerns about eliminating in-person interactions altogether [50]. Consequently, digital consent tools may currently be best suited as preparatory or supplementary instruments rather than as complete substitutes for personal interaction – though this may evolve as AI systems become more reliable and contextually sensitive. A blended approach has already shown promise, with both patients and clinicians reporting positive experiences when digital and verbal information are combined [52, 65], as it preserves direct patient contact and enables clinical assessment on site [50]. Notably, in hybrid formats, trust in the consistency of digital and verbal information is crucial [63]. Looking ahead, the continued evolution of AI may eventually enable highly advanced conversational agents or avatars to conduct parts of the consent process independently. While this could expand access and efficiency, it remains unclear whether such systems can fully meet patients’ emotional and interpersonal needs. Future research should examine whether these technologies can preserve essential aspects of trust and empathy that are traditionally associated with human interaction. Overall, practical and legal barriers persist. For instance, physicians have highlighted unresolved issues around the digital documentation of informed consent, such as the validity of electronic signatures [50, 63]. There is no uniform legal framework for electronic informed consent. A recent global survey found that while some jurisdictions (e.g., the United States, Singapore) explicitly permit e-consent, others allow it only under vague or pilot provisions, and several still prohibit it altogether. In Europe, the legal picture is further complicated by the interplay between the General Data Protection Regulation (GDPR), national e-signature laws (eIDAS), and forthcoming requirements of the EU Artificial Intelligence Act (expected 2025), all of which impose strict transparency, security, and documentation standards. In the United States, the FDA’s 2023 draft guidance on electronic informed consent likewise mandates robust identity verification and audit trails. These layered regulations mean that digital consent platforms, and especially AI-enabled tools that may be classified as software as a medical device, must be adapted to local compliance regimes, making legal uncertainty a genuine barrier reported by clinicians. These insights emphasize that technological innovation alone is insufficient: Successful integration into clinical practice depends on sociotechnical alignment, patient-centered design, and sustained ethical oversight. As technologies rapidly evolve, it is imperative that legal and ethical frameworks develop in tandem to ensure not only regulatory clarity but also safeguard patient autonomy, data protection, and public trust.

### Implications for future research

This review highlights several directions for future research. Given the rapid technological advancement and increasing integration of digital tools into clinical routines, there is a pressing need to evaluate emerging technologies under real-world conditions. Empirical studies conducted in authentic healthcare settings are essential to ensure that findings remain both relevant and transferable. In doing so, it is important to also explore the potential exclusion of patients with limited digital literacy or impairments from digital consent processes. It is essential to investigate how these tools can be made more inclusive and accessible, ensuring that all patient groups can participate equally.

Moreover, the role of healthcare professionals in the context of digital consent processes has so far received limited attention. While clinicians are often conceptualized as facilitators of digital implementation, emerging evidence from related fields suggests that digital technologies, particularly those based on AI, can substantially affect healthcare professionals. For instance, a study by Sumrall et al. [83] demonstrated that an AI-driven scheduling system improved physician satisfaction, reduced burnout, and increased work-life balance, while also leading to fewer adverse events and substantial cost savings in healthcare operations. Future research should therefore examine how digital consent tools can benefit healthcare professionals, with potential implications for improving workflow efficiency and reducing clinician stress in high-pressure environments.

To strengthen the overall evidence base, future studies should increasingly apply experimental or quasi-experimental designs that allow for causal inference. Such designs are particularly valuable for assessing the effectiveness, risks, and unintended consequences of digital consent tools and can inform the development of evidence-based guidelines and regulatory standards.

### Implications for practice

For clinical practice, the findings of this review underscore several critical considerations for the effective and ethically responsible implementation of digital consent technologies. First and foremost, digital tools should be developed with a consistent focus on patient needs and diversity. Systems must be intuitive, accessible, and adaptable to varying levels of technological literacy, while also allowing patients to control the timing and pacing of information delivery. Particular emphasis should be placed on fostering trust through transparent communication of data use and privacy, as well as the consistency between digital and face-to-face information. Involving patients and healthcare staff during the development phase is a key factor for long-term acceptance and usability. Where AI technologies are used to simplify language or adapt content to individual needs, medical oversight is essential to ensure factual accuracy and contextual appropriateness. The successful application of AI in healthcare also depends on the engagement of healthcare professionals, adequate training, and supportive infrastructures [84]. To preserve ethical standards and interpersonal trust, digital consent tools should therefore be embedded in a blended model that supplements – but does not replace – personal interaction, particularly in ethically sensitive contexts. A seamless integration of digital consent tools into existing clinical workflows requires clear procedural guidelines, appropriate training for healthcare professionals, and technical support structures. Persisting legal uncertainties present practical challenges. Aligning legal frameworks with emerging regulations, such as the EU AI Act, may help to address these barriers and support broader adoption. In addition, the cost-effectiveness of digital consent systems should be assessed not only in terms of operational efficiency and data security, but also with regard to their potential impact on documentation quality and patient safety. While initial implementation costs may be substantial, these systems may contribute to reducing administrative workload and improving the overall consent process, provided that ethical, legal, and technical requirements are adequately addressed.

### Limitations

In view of the rapid progress of digitalization, we consider the limitation of this review to articles from the years 2012 to 2024 to be appropriate in order to ensure applicability to current work processes. Despite adherence to the JBI Manual for Evidence Synthesis for scoping reviews [41] and the carefully considered selection of keywords and databases used for the search, the possibility remains that relevant studies may not have been taken into account. This review was limited to studies published in English and German due to the language proficiency of the authors. As a result, relevant studies published in other languages may have been excluded, which could introduce language bias. In addition, grey literature – such as unpublished reports, dissertations, and government documents – was not included in the search strategy. While the focus on peer-reviewed literature ensured a consistent level of methodological transparency, the exclusion of grey literature may have led to an underrepresentation of practical implementation experiences or unpublished findings. In line with the methodological guidance for scoping reviews, we did not perform a graded appraisal of the included studies, as the objective of a scoping review is to map the extent, range, and nature of research activity – regardless of the methodological quality of individual sources – in order to provide an overview of the existing evidence [40, 85]. Instead, we focused on systematically charting and categorizing the available literature. The diversity of study designs presented in Additional file 2 illustrates the variety of methodological approaches employed in this research area. Although no formal quality appraisal was conducted, we observed that only a small number of studies employed experimental or quasi-experimental designs that can, in principle, support causal inference. Especially in light of the ongoing advancement of AI-based tools, expanding the body of knowledge by conducting methodologically rigorous studies is essential.

## Conclusions

This scoping review demonstrates that digital and AI-supported tools can substantially enhance the informed consent process by improving patient comprehension, individualizing information delivery, and streamlining clinical workflows. AI-based systems in particular offer novel possibilities for adapting consent content to individual needs and communication styles. However, these technologies must be carefully evaluated in real-world settings, especially with regard to their accuracy and reliability. It is also essential to consider populations with limited digital literacy or access, who risk being excluded if consent processes become overly reliant on digital tools. Future research should focus on experimental or quasi-experimental designs to test outcomes across different user groups, while critically examining the ethical, legal, and social implications of digital consent. Comparative studies are needed to determine when AI adds clear value over conventional tools and whether it can preserve trust, empathy, and interpersonal connection. Ultimately, digital consent should be implemented as part of a blended, patient-centered approach that complements – not replaces – human interaction and ensures accessibility, safety, and transparency.

## Supplementary Information

Additional file 1. Search string and PRISMA-S Checklist.

Additional file 2. Results of the literature search.

## Data Availability

No datasets were generated or analysed during the current study.
