# Supplementary material for: Digitalizing informed consent in healthcare: a scoping review
Source: BMC Health Serv Res. 2025 Jul 2;25:893. doi: 10.1186/s12913-025-12964-7 (PMC12225439; doi:10.1186/s12913-025-12964-7)
Supplement: Supplementary file 1 — Additional file 1. Search string and PRISMA-S Checklist. [file 12913_2025_12964_MOESM1_ESM.pdf]

Search string and PRISMA-S Checklist

Search string

(„patient information“ OR (consent AND patient))
NOT
(education AND readability)
AND
(chatbot OR “dialogue system” OR “dialog system” OR “digital communication system” OR “digital communication assistant” OR “voice assistant” OR “conversational assistant” OR SIRI OR Alexa OR “Google Assistant” OR “IBM Watson” OR “artificial intelligence” OR AI OR web-based OR online OR NLG OR “natural language generation” OR NLP OR “Natural language processing” OR NLU OR “Natural language understanding”)
AND
(health OR “public health” OR “health care” OR healthcare OR ”health service” OR “health sector” OR radiology OR “clinical radiology” OR “interventional radiology”)

+ date: 01/01/2012 to 31/01/2023 [initial search on 2 February 2023]
+ date: 01/02/2023 to 18/06/2024 [updated search on 18 June 2024]*

*This follow-up search was conducted to update the results, particularly with respect to recently emerged technologies such as ChatGPT.

The following table contains a detailed description of the search methods and information sources in accordance with the Preferred Reporting Items for Systematic reviews and Meta-Analyses literature search extension (PRISMA-S)[1]:

PRISMA-S Checklist

Section/topic	#	Checklist item
INFORMATION SOURCES AND METHODS		
Database name	1	The following electronic databases were searched: APA PsycArticles (EbscoHost), APA PsycInfo (EbscoHost), CINAHL (EbscoHost), Communication Abstracts (EbscoHost), eBook Collection (EbscoHost), eBook Open Access Collection (EbscoHost), MEDLINE (EbscoHost), OpenDissertations (EbscoHost), PSYNDEX Literature with PSYNDEX Tests (EbscoHost), Web of Science Core Collection (Web of Science), MEDLINE (Pubmed), PubMed Central (PMC; Pubmed), Bookshelf (Pubmed), PSYNDEX (PubPsych), PASCAL (PubPsych), ISOC-Psicología (PubPsych), MEDLINE (PubPsych), ERIC (PubPsych), NARCIS (PubPsych), NORART (PubPsych), PsychOpen (PubPsych), PsychData (PubPsych).
Multi-database searching	2	NA
Study registries	3	NA

Online resources and browsing	4	NA
Citation searching	5	NA
Contacts	6	NA
Other methods	7	NA
SEARCH STRATEGIES		
Full search strategies	8	**Web of Science:**<br><br>(((ALL=("patient information" OR (consent AND patient))) NOT ALL=(education AND readability)) AND ALL=(chatbot OR "dialogue system" OR "dialog system" OR "digital communication system" OR "digital communication assistant" OR "voice assistant" OR "conversational assistant" OR SIRI OR Alexa OR "Google Assistant" OR "IBM Watson" OR "artificial intelligence" OR AI OR web-based OR online OR NLG OR "natural language generation" OR NLP OR "Natural language processing" OR NLU OR "Natural language understanding")) AND ALL=(health OR "public health" OR "health care" OR healthcare OR "health service" OR "health sector" OR radiology OR "clinical radiology" OR "interventional radiology")<br><br>*+ manually added date range*<br><br>**EbscoHost:**<br><br>*The databases were selected manually. The search string was entered manually in the fields provided:*<br><br>(„patient information“ OR (consent AND patient))<br><br>NOT: (education AND readability)<br><br>AND: (chatbot OR “dialogue system” OR “dialog system” OR “digital communication system” OR “digital communication assistant” OR “voice assistant” OR “conversational assistant” OR SIRI OR Alexa OR “Google Assistant” OR “IBM Watson” OR “artificial intelligence” OR AI OR web-based OR online OR NLG OR “natural language generation” OR NLP OR “Natural language processing” OR NLU OR “Natural language understanding”)<br><br>AND: (health OR “public health” OR “health care” OR healthcare OR ”health service” OR “health sector” OR radiology OR “clinical radiology” OR “interventional radiology”)<br><br>*+ manually added date range*<br><br>**Pubmed:**<br><br>((((„patient information" OR (consent AND patient))) NOT ((education AND readability))) AND ((chatbot OR "dialogue system" OR "dialog system" OR "digital communication system" OR "digital communication assistant" OR "voice assistant" OR "conversational assistant" OR SIRI OR Alexa OR "Google Assistant" OR "IBM Watson" OR "artificial intelligence" OR AI OR web-based OR online OR NLG OR "natural language generation" OR NLP OR "Natural language processing" OR NLU OR "Natural language understanding"))) AND ((health OR "public health" OR "health care" OR

		healthcare OR "health service" OR "health sector" OR radiology OR "clinical radiology" OR "interventional radiology")) Filters: from 2012/1/1 - 2023/1/31<br><br>**PubPsych:**<br><br>(„patient information“ OR (consent AND patient)) NOT (education AND readability) AND (chatbot OR “dialogue system” OR “dialog system” OR “digital communication system” OR “digital communication assistant” OR “voice assistant” OR “conversational assistant” OR SIRI OR Alexa OR “Google Assistant” OR “IBM Watson” OR “artificial intelligence” OR AI OR web-based OR online OR NLG OR “natural language generation” OR NLP OR “Natural language processing” OR NLU OR “Natural language understanding”) AND (health OR “public health” OR “health care” OR healthcare OR ”health service” OR “health sector” OR radiology OR “clinical radiology” OR “interventional radiology”)<br><br>*+ manually added date range*
Limits and restrictions	9	Due to the rapid development of technologies and preliminary test searches indicating an increasing number of relevant articles in recent years, the search was limited to publications from 2012 to 2024.
Search filters	10	NA
Prior work	11	NA
Updates	12	Two consecutive searches were conducted and limited by publication date, first from 1 January 2012 to 31 January 2023, and again from 2 February 2023 to 18 June 2024. The updated search strategy was consistent with the original search.
Dates of searches	13	A comprehensive literature search was initially run on 2 February 2023 and then rerun on 18 June 2024.
PEER REVIEW		
Peer review	14	The strategies were discussed among the authors.
MANAGING RECORDS		
Total records	15	The search on 2 February 2023 yielded a total of 9699 records (Web of Science: 2.181, Ebsco Host: 2.847, Pubmed: 3.820, PubPsych: 851), of which remained 3250 after duplicate removal. The search on 18 June 2024 yielded a total of 2072 records (Web of Science: 626, Ebsco Host: 647, Pubmed: 708, PubPsych: 91), of which remained 1037 after duplicate removal. This results in a total number of 4287 unduplicated records eligible for title and abstract screening.
Deduplication	16	Duplicates were removed using citation management software Zotero.

[1] Rethlefsen ML, Kirtley S, Waffenschmidt S, Ayala AP, Moher D, Page MJ, et al. PRISMA-S: an extension to the PRISMA Statement for Reporting Literature Searches in Systematic Reviews. Syst Rev. 2021; 10: 39. doi: 10.1186/s13643-020-01542-z.
