# Supplementary material for: Digitalizing informed consent in healthcare: a scoping review
Source: BMC Health Serv Res. 2025 Jul 2;25:893. doi: 10.1186/s12913-025-12964-7 (PMC12225439; doi:10.1186/s12913-025-12964-7)
Supplement: Supplementary file 2 — Additional file 2. Results of the literature search. [file 12913_2025_12964_MOESM2_ESM.pdf]

Additional file 2. Results of the literature search

	Method	Target group & context	Technology (and intervention) *(Results related to RQ1)*	Design & sample	Evaluation results *(Results related to RQ2)*	Implementation success factors *(Results related to RQ3)**
**Technology not specified or telemedicine in general**						
Coppola et al. [46]	Quant	Physicians; radiology; Italy	Telemedicine, not specified	Cross-sectional survey; *N* = 1791 radiologists	• *Perceived advantages of patient's informed consent (PIC) dematerialization:* easier and faster PIC recovery (96·5%), safer storage and conservation (94·5%), reduced running costs (90·7%)<br>• *Potential disadvantages:* need to create dedicated areas for PIC acquisition inside each radiological unit (64%), preliminary approval for the use of advanced digital signature tools from patients (51·8%)<br>• Overall, 94·5% of respondents had a positive opinion about PIC dematerialization.	• In order to maintain legal validity, features of conventional paper-based PIC acquisition should be incorporated in digital PIC, such as the possibility for patients to revoke their consent at any time before radiological procedures.
Kassam et al. [47]	Review	Patients; various fields and countries	Telemedicine, not specified	Structured literature search including primary peer-reviewed articles from January 2010 to March 2022	91% of the studies found that participants were willing to provide consent, but their consent behaviors and preferences were context dependent.	• Information must be customizable, allowing readers to tailor the granularity of detail to their individual needs.<br>• Transparency in these processes is crucial for building trust in digital health tools and utilizing data innovatively to improve health and system outcomes.<br>• Common requirements for patient consent included clear and understandable information about who has access to their personal health information (PHI), for what purpose their PHI will be used, and how their privacy will be protected.<br>• In 71% of the studies, participants preferred granular, informative, and transparent consent choices.

Mirza et al. [48]	Review	Patients; surgery with focus on neurosurgery; UK	Telemedicine, not specified	Systematic review and meta-analysis	• Electronic technologies significantly enhanced patient satisfaction with the informed consent process and patients' gain in knowledge compared to standard nonelectronic practices.<br>• Neurosurgical patient knowledge was significantly enhanced with electronic technologies when compared to other surgical patients.<br>• No difference in patient satisfaction between neurosurgical cohorts and other surgical patients with respect to electronic technologies.	The most appropriate type of electronic consent technology will vary according to the surgical procedure and patient circumstances; individual health services must design tailored consenting interventions based on patient preferences and resources
Kolcun et al. [49]	Review	Patients; spine surgery; USA	Telemedicine defined as "any form of intervention or communication via online, digital, or phone-based platforms between spine surgery patients and their surgeons or clinical staff" (p. 162)	Systematic review including studies from 2016 to 2019	• Use of TM for certain tasks such as postoperative surveys was preferred by patients for convenience, over in-person options.<br>• A mobile app for preoperative instructions and reminders can increase patient compliance and decrease case cancellation.<br>• A multi-media online educational tool for preoperative education regarding disease and surgical consent can improve patients' understanding of disease, surgical approach, prognosis, and postoperative course.	• Clinical applications of TM have predominately focused on enhancing perioperative communication between patients and their surgeons.
Neumann et al. [50]	Quant	Practitioners; (pediatric) anesthesia; Europe	Telemedicine, not specified	Cross-sectional survey; $N = 930$ members of the European Society of Anesthesia and Intensive Care Medicine (ESAIC) in 47 European countries (920 medical doctors, 2 nurses, 6 physician assistants, 1 other)	• *Possible advantages of remote interviews*: limit waiting time for interview, more efficient than a face-to-face interaction, less stressful, use of standardized questionnaires as an advantage from an organizational perspective<br>• *Concerns*: lack of contact, missing out on personal observation of the patient, missing doctor-patient relationship, legal uncertainty	• For the majority of participants, it is not possible to obtain informed consent via the Internet in a routine setting.<br>• Remote informed consent may not comply with legal regulations (major differences across European countries).<br>• For pediatric practice, depending on complexity of surgeries, informed consent could be given with only one parent present.<br>• Only few participants report special regulations during the pandemic situation favoring online or telephone-based informed consent

Neumann et al. [51]	Quant	Practitioners (mostly medical doctors); anesthesiology and intensive care; Europe	• Telemedicine, not specified<br>Online survey with a total of 27 multiple choice questions	Cross-sectional survey; $N = 930$ members of the European Society of Anesthesiology and Intensive Care from 47 European countries (920 medical doctors, 2 nurses, 6 physician assistants, 2 undisclosed professionals)	NA	European countries rarely use remote informed consent, mainly for repeated anesthetic procedures, and possibly due to inconsistent legal and technical structures across Europe:<br>• 23.1%/37.2% of the participants indicated that it was possible to obtain consent online/via telephone (more often reported in countries with high than in countries with low gross domestic product per capita (GDPPC) levels); an improvement in technical standards in poorer countries could lead to wider use of digital media and thus benefit certain patient groups.<br>• Most respondents would prefer to obtain informed consent in person in the future (high GDPPC: 75.1%, middle GDPPC: 83.6%, low GDPPC: 79.1%).<br>• Large proportion of respondents were unclear about the legal regulations (35–39%), indicating widespread uncertainty regarding legal frameworks; there is a necessity of a clear legal framework within Europe that could be beneficial for patients and involved anesthesiologists.<br>• The patient was mostly informed at least one day before the procedure (in 67.1% of cases for simple procedures and in 85.2% of cases for complex procedures). Information via internet could enable adequate lead time for the potential pre-operative improvement of the patient's health.

Kaller et al. [52]	Quant	Patients; abortion care; USA	Video conference<br>Pre-abortion informed consent through telemedicine vs. in person (note: in person setting generally in groups)	Cross-sectional survey; $N = 383$ patients seeking abortion ($n_{telemedicine} = 166$, $n_{in\ person} = 217$)	• Telemedicine participants had higher odds of being "very satisfied" with the visit and "very comfortable" asking questions during the visit compared to participants who attended in-person visits.	• Telemedicine participants would have had to travel significantly further than in-person participants traveled to attend the visit at the clinic; authors recommend telemedicine as an alternative to in-person options to overcome barriers, such as childcare, travel costs/time, and missed work.
**Video**						
Berner et al. [53]	Quant	Patients; lumbar puncture surgery; Great Britain	• Patient information video that covers the journey of a patient who undergoes lumbar punctures and addresses the risks and benefits of the procedure using non-jargon language<br>• Two groups (verbal explanation and video observation vs. video only)	Pre-post survey; $N = 11$ ($n_{verbal+video} = 5$, $n_{video\ only} = 6$)	• After watching the patient information video, participants reported significantly higher subjective understanding of the procedure and satisfaction with questions being sufficiently answered.<br>• No significant difference in understanding, recall, and satisfaction between the two groups	• Authors conclude that audiovisual tools can be a valuable addition to the consent process, especially with regard to standardization of information.
Wald et al. [54]	Quant	Patients; cardiology; Great Britain	• Patient information video: multi-language animations explaining angiography and angioplasty<br>Patients were recorded either after or before introduction of the animations into practice, resulting in two groups (animation group vs. no animation group)	Nonrandomized control group design; $N = 200$ patients with acute coronary syndrome before urgent transfer to a cardiac center for their procedure ($n_{animation} = 100$, $n_{no\ animation} = 100$)	Compared to patients who did not watch the multi-language animations, patients who did reported significantly higher understanding of the reason for transfer, the procedure, its benefits, and risks.	• *Implementation facilitators*: presenting initiative at district hospital governance meeting at outset, 'prescribing' the animations on the consultant ward round, use of videobooks on wards without internet, use of weblink and QR codes on wards with internet, adding animation to inter-hospital transfer checklist, adding animation initiative to junior doctor induction<br>• *Implementation barriers*: patients admitted to noncardiac wards without videobooks, language barriers<br>• *Further ideas*: increased number of videobooks on outlying wards, printing QR codes (linking to animation) on junior doctor daily patient lists, increasing range of

						languages available, periodic central audit of uptake fed back to referring hospital
Cheung et al. [55]	Quant	Patients; clinical pathology (blood transfusion); Canada	• 9-minute video about the benefits of, risks of, and alternatives to transfusion<br>Written survey after consent discussion; then video viewing; then survey about evaluation of the video and willingness to consent to blood transfusion	Pre-post survey, no control group; $N = 25$ patients receiving their first transfusion	• After the traditional consent discussion (before watching the video), there was a high variability in the information recollected by patients, which led to one quarter of patients attempting to obtain additional information about transfusion, primarily from the internet.<br>• After watching the video, patients reported higher subjective understanding of the risks, benefits, and alternatives to transfusion, but no change in comfort with blood transfusion consent.	NA
Moussa et al. [56]	Qual	Patients; adjuvant radiation therapy (RT) for early breast cancer; Australia	• 18-minute video addressing a 30-item knowledge of RT scale; filming took place at the same locations as the radiation therapy; Virtual Environment for Radiotherapy Training (VERT) software was used for simulations<br>After completing a brief questionnaire, participants viewed the video online before 30-minute focus group discussions, using prompts to reflect on the video's effectiveness.	Focus groups; $N = 10$ woman with early breast cancer scheduled to receive standard adjuvant radiation therapy after breast-conserving surgery; mean age was 62 years	• Most participants felt the video improved their understanding of RT, appreciating its detailed, step-by-step explanation and visualization of the linear accelerator. While most found the content clear, two noted the explanation of the weekly RT schedule was too fast. The video was seen as a valuable complement to other educational resources.<br>• Participants felt the video reduced anxiety and increased preparedness for RT, with some wishing they had seen it earlier. Most felt their concerns about RT decreased or remained unchanged, though one participant was more concerned about side effects. While the video answered most questions, some uncertainties remained about COVID-19 during treatment, missed sessions, treatment at other hospitals, and post-treatment follow-up.	• Participants appreciated the graphics, found the video length satisfactory, and felt the language was appropriate. While most thought the pace was suitable, some felt parts were too fast and suggested multiple viewings.<br>• All participants positively received the video and would recommend it to other women starting RT. They suggested adding statistics on RT efficacy and providing external access to the video for sharing with family.
Book et al. [57]	Quant	Patients and their families; pediatric surgery (inguinal hernia	• 6-minute video showing the consent dialog<br>Face-to-face consenting in clinic with (intervention) vs. without (control) access to the online video; parents completed survey during their child's treatment	RCT with only one measurement; $N = 50$ parents of children diagnosed with an inguinal	• The intervention group showed significantly higher anxiety and knowledge but no difference in satisfaction compared to the control group.<br>• Of those randomized to the online video group, 68% of participants would recommend watching a supplemental consent video to	NA

		surgery); Germany		hernia in the ambulatory clinic and scheduled for an elective repair ($n_{intervention}$ = 22, $n_{control}$ = 28)	parents with children scheduled for inguinal hernia repair.	
Robertson et al. [58]	Mixed (quant/qual)	Patients; pediatric malignancy biobank; Australia	• 180-second video<br>After watching the video, patients and health professionals rated the video quantitatively and qualitatively regarding its effectiveness in conveying information and supporting consent.	Cross-sectional survey; $N = 31$ (15 patients or caregivers who had previously consented to be part of the biobank; 16 health professionals who were involved in obtaining consent); average age of the patient at consent to the biobank was 8.7 years	• Most patients/caregivers felt informed at consent but overwhelmed and did not engage with the written information.<br>• Most patients/caregivers agreed the video provided enough information to consider participation.<br>• Both patients/caregivers and health professionals rated the video favorably for its information and format, with participants explicitly valuing the video's simplicity and clarity.<br>• Health professionals found the video effective as a conversation starter to formalize written consent but noted the need for linguistic translations to better support families.	• The video appears particularly valuable as a precursor to an interactive, formal consent discussion, not as a replacement of traditional consent information sheets or discussions.
**Website**						
Fraval et al. [59]	Quant	Patients; elective orthopedic surgery; Australia	• Patient education webpage; designed as a walkthrough overview of each procedure along the lines of diagnosis and indications for surgery, complications, pre-operative, intra-operative, and post-operative care<br>• Standard discussion with the treating surgeon (control) vs. discussion and additional online education webpage (intervention)	RCT with only one measurement; N = 211 patients undergoing one of five common orthopedic procedures ($n_{intervention}$ = 103, $n_{control}$ = 108)	Compared to the control group, the intervention group showed significantly higher patient knowledge and satisfaction, but no difference was found relating to their anxiety scores.	NA

Zevin et al. [60]	Quant	Patients; surgery; Canada	• Digital education platform (DEP): 29-slide video-supplemented module detailing the risks, benefits, expectations, and outcomes for the laparoscopic Roux-en-Y gastric bypass (LRYGB)<br>Standard verbal consent (control) vs. verbal consent and additional DEP (intervention)	RCT; pre-post-follow up; N = 51 candidates for a LRYGB ($n_{intervention}$ = 26, $n_{control}$ = 25)	• Compared to the control group, the intervention group showed significantly higher post-consent knowledge and shorter duration of time to obtain informed consent, but no difference was found in patient satisfaction and knowledge retention at four to six weeks.<br>• 97% of participants in the intervention group were satisfied with the online DEP module.<br>• The addition of a DEP online module to a standard verbal consent for LRYGB resulted in over 50% time savings for the bariatric surgeon conducting the consent discussion.	Time savings for clinicians through structured digital education module
**Digital questionnaire**						
Goodhart et al. [61]	Quant	Patients; surgery; USA	Electronic Personal Assessment Questionnaire PreOperative (ePAQ-PO); designed for clinical<br>use in preoperative assessment for patients with different clinical conditions and requirements	Questionnaire (with retest) and interrater-reliability by comparing self-reports with expert-ratings; N = 300 patients scheduled for surgery in different medical fields	Overall, ePAQ-PO has the potential to improve the efficiency and accuracy of preoperative assessments.	• ePAQ-PO was acceptable to patients and the data collected using the system were reliable.<br>• The intrinsic scoring systems for American Society of Anesthesiologists physical status and Body Mass Index in ePAQ-PO were comparable with values assigned by clinicians.
**Interactive web application**						
Dathatri et al. [62]	Quant	Patients; surgery; USA	• 20- to 40-minute interactive web-based audiovisual presentation containing relevant graphics describing standard informed consent components regarding coronary angiography and percutaneous coronary intervention (PCI)<br>• Scripted verbal or written consent process (group 1) vs. web-based, audiovisual presentation (group 2)	Randomized pre-post pilot study; N = 102 patients undergoing first-time elective, outpatient cardiac catheterization and possible PCI ($n_{group\ 1}$ = 48, $n_{group\ 2}$ = 54)	• Changes in patient comprehension rates were similar between groups for risk and benefit consent elements, but group 2 had significantly greater improvement in the identification of treatment alternatives (it should be noted that the digital consent tool provided more information on this than the written consent).<br>• In both groups, correct identification of all risks and alternatives increased significantly after consent.<br>• Independent of informed consent approach, misperceptions of benefits persisted after consent.	NA

Assale et al. [63]	Mixed (quant/qual)	Patients and practitioners; Italy	• Prototype of an interactive web-based application for informed consent called Digital Informed Consent Experience (DICE); patients can demand a definition by clicking on technical terms or react to given information with emoticons, triggering the system to send a notification to the doctor (if *I don't understand this*) or nurse (if *I feel worried*)<br>• Standard questionnaire-based usability study involving representative users who were previously involved in some user tests (not reported in this study); followed by a qualitative user study with key informants who could represent the main stakeholders involved in the informed consent process	Questionnaire-based, cross-sectional usability study with $N = 24$ prospective users; series of individual semi-structured interviews with $N = 6$ stakeholder represents ($n_{patients} = 2$, $n_{physicians} = 2$, $n_{nurses} = 2$)	• High recommendability (in pertinent situations) for the DICE prototype: 14 promoters (58%), 7 passives (29%), and 3 detractors (13%)<br>• Users would not feel excessively embarrassed or uncomfortable in expressing either lack of understanding nor concerns through the system.<br>• Mix of web-based and paper due to legal obligation of having the informed consent be signed on paper time<br>• A computer system like DICE could make it easier to express incomprehension.<br>• Giving the patients the capability to say "I am worried" for each paragraph of the informed consent could make them more anxious, not less which might lead to additional load for nurses.	• Men perceived the prototype as significantly less attractive than the female respondents.<br>• The application was found to be equally intuitive by expert users and those who claimed to use web applications infrequently.<br>• Professional participants (nurses and physicians) expressed a concern regarding the lack of time in hospital work; also the patients noticed that relatively little time was spent on reading and signing the informed consent.<br>• Necessary skills that the patients should have to use such an application like DICE could be a problem, especially for elderly patients.<br>• Legal obligation of having the informed consent be signed on paper could lead to cumbersome<br>• An application as DICE would require strong modifications in the routine and pre-surgery workflow.
Siracuse et al. [64]	Qual	Patients and physicians; surgery; USA	• Web-based consent and booking system; integrated into an electronic booking program; available via intranet; after the patient and the procedure are selected, a defined personalized template is available to the surgeon<br>• Interactive implementation process including a qualitative survey phase with possible end users to identify key requirements of the system to be developed	Three design and implementation phases (survey phase, design and implementation phase, post implementation and revision phase); N = NA ("various stakeholders")	*Results from the implementation phase:*<br>• Consent forms need to be printed out for signing, resulting in additional load for practitioners.<br>• Electronic consent more complete than paper-based (e.g. time notations)<br>• No lost consent forms (vs. 20% prior to implementation)<br>• Elimination of illegibility problem<br>• Saves time for administrative assistants	*Survey with end users:*<br>• Minimal free text was desirable.<br>• Different database fields were required by different users, therefore a user's view should be tailored to his or her specific role and based on his or her unique system login.<br>• A single template for a procedure or consent for all surgeons would not achieve user buy-in.<br>• Templates should be editable.<br>• Booking should be editable for the specific needs of patients.<br>• Because one surgeon worked remotely from their support staff, a

						booking initiation notification would have to be created.<br>• Diagnostic and procedure codes should be expressed in different ways, depending on the user (professional terminology for staff, plain language for patients).<br>• Booking information should be available to the operating room and central supply personnel to facilitate procedure-specific equipment and room setup, as well as surgeon-specific preferences.
**AI**						
Schmidlen et al. [65]	Qual	Patients; genetic counseling; USA	• Consent chatbot that walks patients through components of the consent allowing them to opt to receive more or less detail on key topics (goals, benefits, risks, etc.) [cascade chatbot and family sharing chatbot not further considered in this review]<br>• Three focus groups reviewed the consent chatbot	Cross-sectional study; $N = 33$ patients with current active enrollment in MyCode®	• Higher learning gain than in conventional conversation<br>• High ease of use<br>• Convenient to go through the chatbot conversation in own time and pace	• *Concerns*: access to and comfort with this kind of technology; therefore important to continue to utilize the current in-person consent approach, as this method would still capture those without reliable internet or smartphone access and those who are hesitant to use chatbots<br>• The consent chatbot presents an engaging alternative to deliver content challenging to comprehend in traditional paper or in-person consent; participants supported the use of chatbots to consent for genomics research (despite having limited familiarity with chatbots prior to the focus groups).<br>• Little importance of chatbot's name and avatar image (personification perceived as positive)
Ali et al. [66]	Mixed (quant/qual)	Patients; surgery; USA	• ChatGPT-4 (AI-based Chatbot by OpenAI)<br>Generic surgical consent forms and procedure specific consent forms were simplified using	Cross-sectional study; $N = 9$ (3 physician authors, 1 malpractice defense	GPT-4-assisted simplification of *15 generic surgical consent forms* with confirmed medical and legal sufficiency consistency led to…<br>• …a significant reduction in average reading time, word rarity, and passive sentence frequency	NA

			GPT-4; results were rated before and after	attorney, 5 subspecialty surgeons) with a total of 20 consent forms (15 surgical consent forms and five consent forms for diverse surgical procedures)	• …a significant improvement of readability (from an average college freshman to an 8th-grade level, matching the average American's reading level)<br>Expert review by subspecialty surgeons of *five procedure-specific consent forms* for varied surgical procedures transformed by GPT-4 to an average 6th-grade reading level yielded no wording changes or significant clinical inaccuracies requiring correction	
Shiraishi et al. [67]	Quant	Patients; plastic surgery; Japan	• ChatGPT (AI-based Chatbot by OpenAI); accessed September 2023<br>Two prompts were created to generate consent documents for levator advancement surgery; responses from ChatGPT and the original document were evaluated for accuracy, informativeness, and accessibility.	Cross-sectional study; $N = 8$ practitioners (4 board-certified plastic surgeons and 4 nonmedical staff members)	• Board-certified plastic surgeons rated the initial AI-generated IC document lower than the original in accuracy, informativeness, and accessibility. The revised AI-generated document (second prompt, including information on what content to include in the consent form) also scored lower in accuracy and accessibility. Nonmedical staff found no significant differences between the AI-generated and original documents in these aspects.	• Current ChatGPT cannot serve as a standalone patient education resource, but it has potential for creating better IC documents with improved professional terminology.
Currie et al. [68]	Quant	Patients; nuclear medicine; Australia	• ChatGPT-3.5 and ChatGPT-4 (AI-based Chatbot by OpenAI); accessed between April and June 2023<br>• ChatGPT was employed to create patient information sheets for informed consent in 7 nuclear medicine procedures. Sheets were generated separately using GPT-3.5 and GPT-4 architectures, then evaluated based on accuracy, appropriateness, currency, and suitability.	Cross-sectional study; $N = 3$ expert nuclear medicine technologists or scientists	• GPT-3.5 provided patient information suitable for patients but lacked accuracy, currency, and omitted important details, rendering it unfit for purpose.<br>• GPT-4 improved patient information in terms of appropriateness, accuracy, and currency, although it still had some omissions.<br>• While GPT-3.5 offers accessible and generally plausible patient information, its inaccuracies and omissions pose risks for patients and informed consent. In contrast, GPT-4 is more accurate and suitable for its intended use.	NA

Kienzle et al. [69]	Quant	Patients; knee arthroplasty; Germany	• ChatGPT-4 (AI-based Chatbot by OpenAI); accessed June 2023<br>• Common patient questions were presented to ChatGPT; accuracy was validated, and responses were independently rated	Cross-sectional study; $N = 3$ orthopedic surgeons with a total of 49 questions	• The scores assigned to ChatGPT's responses predominantly surpassed a threshold of three out of five.<br>• The content itself demonstrated commendable precision and reliability.<br>• However, 37% of the references associated with ChatGPT's responses were fabricated, 15% were referenced with the correct digital object identifier and/or PubMed ID; only 44% aligned seamlessly with the context depicted in the responses; there is critical need for validation and ongoing evaluation of information from external, reputable sources, particularly in the context of clinical decision making and patient education	NA
Stroop et al. [70]	Quant	Patients; spinal surgery; Germany	• ChatGPT (AI-based Chatbot by OpenAI); accessed February 2023<br>• Surgeons were instructed to use ChatGPT to inform themselves about acute lumbar disc herniation (LDH) from a layman's perspective; then they rated the quality of ChatGPT's answers.	Cross-sectional study; $N = 24$ spinal surgeons with a total of 139 questions to ChatGT	• Overall, ChatGPT provided good results in comprehensibility, specificity, satisfaction of responses, medical accuracy, completeness.<br>• However, ChatGPT tended to be more general, requiring patients to have some prior knowledge to ask specific questions or to repeat the question in a more specific way.<br>• Although ChatGPT offered information that went beyond the details contained in the declaration of consent, it could not cover all details.<br>• Occasionally, ChatGPT made minor inaccuracies, like mentioning kyphoplasty and vertebroplasty as LDH treatments.<br>• The lack of a graphical presentation was considered to be a distinct disadvantage of ChatGPT.	NA
Moll et al. [71]	Qual	Patients; radiotherapy ; Austria	• ChatGPT-3.5 (AI-based Chatbot by OpenAI); Voice Control for ChatGPT freeware was used to create a conversational environment (speech-to-text for input)<br>• The patient discussed her treatment with ChatGPT 3.5,	Cross-sectional study; $N = 1$ woman diagnosed with breast cancer (professional background in	• *Correctness of information provided by ChatGPT:* Most answers provided were correct, but some, like loss of scalp hair, were incorrect. ChatGPT also omitted certain side effects.<br>• *Perception of the patient regarding the use of ChatGPT:* The patient found ChatGPT acceptable but preferred a physician present, as	Preference to engage with a physician rather than a chatbot in debriefing emphasizes the continued significance of human interaction in healthcare settings.

			under a physician's supervision, followed by a final evaluation and testing reproducibility over ten iterations in three weeks.	the field of informatics)	she mistrusted ChatGPT due to its nontransparent information sources.<br>• In addition to the free ChatGPT-3.5 model, the premium 4.0 model was tested, yielding generally similar answers (no additional side effects or countermeasures were mentioned), with superior handling of conversational nuances.	
Aydin et al. [72]	Quant	Patients; coronary angiography (CAG); Turkey	• ChatGPT-3 (AI-based Chatbot by OpenAI)<br>• Participants randomly assorted to the study group received informed consent by asking questions to the chatbot; in the control group, doctors explained CAG using a standard consent form and answered questions.	RCT; $N = 139$ patients undergoing CAG for the first time ($n_{\text{study group}} = 70$, $n_{\text{control group}} = 69$)	• Informed consent obtained with the AI chatbot was as accurate and comprehensive as the conventional method.<br>• The correct understanding of CAG risks was significantly higher in the study group compared to the control group, but there were no differences in satisfaction.<br>• Patients using the AI chatbot might have received more accurate and personalized information about CAG risks, as they could ask questions freely and read responses at their own pace, aiding in better understanding and informed decision-making.	NA

RQ = research question, Quant = quantitative study, Qual = qualitative study, N = total number of units in the sample under study, n = number of units in a subgroup of the sample under study, NA = not applicable, RCT = randomized controlled trial, TM = telemedicine, *Design-related implementation factors (e.g., usability, customization) that conceptually relate to both RQ1 and RQ3 are categorized under RQ3 due to their reported relevance for clinical integration or user acceptance.
